# Enteral Feeding in Patients With Open Abdomen and Negative Pressure Therapy: A Propensity Score Analysis

**DOI:** 10.3389/jaws.2024.13702

**Published:** 2024-12-18

**Authors:** Laurent Petit, Nicolas Faure, Bruno Pereira, Vincent Dubuisson, Xavier Berard, Matthieu Biais, Cédric Carrié

**Affiliations:** ^1^ Anesthesiology and Critical Care Department, Pellegrin University Hospital, Bordeaux, France; ^2^ Biostatistics Unit, Délégation Recherche Clinique and Innovation, CHU Clermont-Ferrand, Clermont-Ferrand, France; ^3^ Department of Visceral Surgery, Bordeaux University Hospital, Bordeaux, France; ^4^ Department of Vascular Surgery, Bordeaux University Hospital, Bordeaux, France; ^5^ University Bordeaux Segalen, Bordeaux, France

**Keywords:** nutritional support, enteral nutrition, open abdomen, negative pressure therapy, intensive care

## Abstract

**Introduction:**

In critically ill surgical patients treated with open abdomen and negative pressure therapy (OA/NPT), the association between nutritional support and clinical outcome is still controversial. The main objective of this study was to assess the effect of enteral nutritional support during the acute phase (i.e., the first 7 days) on clinical outcome (abdominal wall closure rate or fistula formation) in critically ill surgical patients treated by OA/NPT.

**Methods:**

Over a 5-year period, every critically ill patient who underwent nutritional support while treated by OA/NPT was retrospectively included. The main study outcome was a composite criterion, defined as delayed abdominal closure ≥8 days and/or secondary abdominal complications (secondary anastomotic leak, intra-abdominal abscess and fascial dehiscence). Inverse probability of treatment weight (IPTW) was derived from a propensity score model. Multivariable logistic regression was used to test the association between clinical outcome and different modalities of nutritional support (enteral nutrition vs. nil per os during the first week after OA/NPT, early vs. late enteral nutrition, normal vs. low caloric/protein intake).

**Results:**

Over the study period, 171 patients were included and 50% underwent delayed abdominal closure and/or secondary abdominal complications. The rate of delayed abdominal closure or secondary abdominal complications was significantly lower in patients who received enteral nutrition versus those who remained nil per os (40% vs. 61%, *p = 0.007*), with an IPTW-adjusted OR of poor clinical outcome of 0.49 [95%CI: 0.25–0.98]. There was no other statistical association between modalities of nutritional support and the study outcome.

**Conclusion:**

In critically ill patients with OA/NPT, the use of enteral feeding within 7 days after surgery was associated with better clinical outcome. Further studies are mandatory to better define the adequate timing for enteral feeding, the energy needs and the protein requirements during the acute phase after OA/NPT.

## Introduction

For several years, open abdomen associated with negative pressure therapy (OA/NPT) has become one of the leading strategies to treat or prevent intra-abdominal hypertension in critically ill patients after a wide range of trauma and non-trauma surgical conditions [1]. However, its use remains controversial as delayed abdominal closure has potential for severe side effects while increasing resource utilization (especially entero-atmospheric fistula and frozen abdomen). Early abdominal closure is thus of paramount importance in order to reduce mortality, complications and length of stay linked to the OA/NPT [2].

In critically ill patients with OA/NPT, optimizing nutrition still represents a complex challenge due to uncertainties regarding the energy needs at different phases of the metabolic response [3]. On the one hand, critically ill patients with OA/NPT are characterized by hyper-metabolic conditions and significant nitrogen loss, which should justify high caloric and protein requirements [4]. In this context, the prompt introduction of enteral nutrition is thought to improve fascial closure rates and reduce infectious complications in patients with OA/NPT [5–7]. On the other hand, other authors reported conflicting findings and rather recommend hypocaloric nutrition given the risk of refeeding syndrome in the early phase of acute illness [8, 9]. Finally, little evidence is available to define the adequate energy and protein intake during the acute phase of illness [10].

The main objective of this study was thus to assess the effect of enteral nutritional support during the acute phase (i.e., the first 7 days) on clinical outcome (abdominal wall closure rate or fistula formation) in critically ill surgical patients treated by OA/NPT. The secondary objective was to determine the adequate timing (i.e., within or after the 48 first hours after OA/NPT) and define the adequate energy and protein intake during the acute phase of critical illness in patients with OA/NPT.

## Methods

### Study Design, Population and Settings

This retrospective cohort study was conducted in a 25-bed Surgical and Trauma Intensive Care Unit (ICU) of a University Hospital (CHU Pellegrin, Bordeaux, France). Over a 5-year period (2015–2020), were eligible for inclusion every adult patient treated by OA/NPT with an ICU length of stay ≥8 days after surgery. During this period, nutrition-related parameters were daily collected into a large ICU database as part of the standard care in every critically ill patient. Patients were excluded when data on nutritional provision were incomplete. The study protocol was declared to the Data Protection Officer in accordance with the French legislation. The observational character of the present study was confirmed by the Institutional Review Board (*IRB*). The patients and/or next of kin were informed about the potential inclusion of their anonymized data for retrospective studies, and none expressed opposition.

Management of medical nutrition therapy was consistent with the up-to-date recommendations [10]. Briefly, enteral nutrition (EN) was introduced as soon as possible in presence of viable and functional gastrointestinal tract after hemodynamic stabilization [5, 6]. Enteral nutrition was delayed in patients with an intestinal tract in discontinuity (temporarily stapled stumps) or in situations of a high output fistula with no possibility to obtain feeding access distal to the fistula or with signs of intestinal obstruction [2]. The caloric delivery was progressively increased up to 80%–100% of estimated needs, determined by adjusted weight-based predictive equations. A theoretical target ≥20 kcal/kg/day was considered adequate during the acute phase (i.e., the first 7 days) following surgery. Parenteral nutrition (PN) was implemented when enteral feeding was contraindicated, if patients did not tolerate EN or when patients did not meet their nutritional targets within five to 7 days. The caloric targets were updated by our dedicated dietician nutritionist, depending on adjusted body-weight, previous nutritional status and ICU-related medical conditions [10]. The caloric targets were lowered and increased progressively in patients at risk of refeeding syndrome (RFS), defined as poor nutritional status previous admission (weight loss >10% within 6 months, BMI <20, ongoing oncological disease, chronic infectious disease or malabsorption syndrome … ) or a starved state >48 h before introduction of nutritional support [11].

### Data Collection, Definitions and Outcomes

For each patient, the following variables were retrospectively collected in the ICU medical record: demographic data, body mass index (BMI) and adjusted body weight (ABW), comorbidities (according to Charlson comorbidity index), poor nutritional status before admission (weight loss >10% within 6 months, BMI <20 kg/m^2^, ongoing oncological disease, chronic infectious disease or malabsorption syndrome), Simplified Acute Physiology Score (SAPS) at ICU admission, modalities of surgical management (type of initial surgery [emergency or scheduled surgery, traumatic or non-traumatic surgery], time and indication for OA/NPT [prophylactic OA or secondary abdominal complication after initial surgery], use of mesh-mediated fascial traction, number of surgical revisions and total duration of OA/NPT before definitive fascial closure).

Caloric intakes, including calories from propofol or glucose infusion, were monitored daily and averaged during the first week after OA. Mean percentage of energy target achievement and cumulative energy deficit were calculated according to a theoretical target ≥20 kcal/kg/day. Were also collected the time of initiation of EN and PN, the use of parenteral omega-3 fatty acid (FA)-containing lipid emulsion, the cumulated protein, glucose and lipid intakes. The occurrence of refeeding hypophosphatemia was defined as a drop of phosphatemia below 0.65 mmol/L within 72 h of the commencement of nutritional support [11]. Temporarily contraindications of EN (temporarily closed loops, gastric aspiration, bowel ischemia, bowel obstruction, high-output fistula, uncontrolled shock or upper GI bleeding) and other ICU-related conditions were also recorded over the first week after OA/NPT (maximal SOFA [Sequential Organ Failure Assessment] score, use of continuous renal-replacement therapy [CRRT], cumulated fluid balance and early use of epidural analgesia).

The main study outcome was a composite criterion, defined as delayed abdominal closure ≥8 days after OA/NPT and/or secondary abdominal complications after OA/NPT (secondary anastomotic leak, intra-abdominal abscess and fascial dehiscence) [5]. Secondary study end-points were the total duration of OA/NPT before definitive fascial closure, the time before transit recovery after OA/NPT, the duration under mechanical ventilation after OA/NPT, the occurrence of ventilator-acquired pulmonary infections (VAP) after OA/NPT, the in-hospital mortality and the ICU or hospital lengths of stay. The impact of nutritional support on outcome measures was assessed, considering different groups of patients (Table 1).

**TABLE 1:** Definitions used for nutritional support and clinical endpoints in critically ill patients with OA/NPT.

Early enteral nutrition	Any patient receiving enteral feeds of at least 10 mL/h and/or 200 kcal/kg within the 48 first hours after OA/NPT
Early Parenteral Nutrition	Any patient receiving parenteral nutrition of at least 10 mL/h and/or 200 kcal/kg within the 48 first hours after OA/NPT
Hypocaloric regimen (underfeeding)	Mean caloric intake <70% of theoretical target over the first week after surgery (theoretical caloric target = 20 kcal/kg/day)
Overfeeding	Mean caloric intake >110% of theoretical target over the first week after surgery (theoretical caloric target = 20 kcal/kg/day)
Low protein diet	Mean protein administration <0.5 g/kg/day over the first week after surgery
High protein diet	Mean protein administration >1.2 g/kg/day over the first week after surgery
Good clinical outcome	Early abdominal closure (i.e., ≤ 7 days) and no intra-abdominal complication after OA/NPT (secondary anastomotic leak, intra-abdominal abscess, and fascial dehiscence)
Poor clinical outcome	Delayed abdominal ≥8 days after OA/NPT and/or secondary abdominal complications after OA/NPT (secondary anastomotic leak, intra-abdominal abscess and fascial dehiscence)

All the definitions and terminologies are in accordance with the recent ESPEN terminology recommendations [10].

### Statistical Analysis

Results are expressed as mean ± standard deviation or median (25%–75% interquartile range) for continuous variables and as absolute or relative frequencies for categorical variables. The data distribution was analysed by a Shapiro-Wilk test. Univariate analysis was first conducted to assess the association between modalities of nutritional support and clinical outcome. Comparisons between continuous variables were performed using the Student t test or the Mann–Whitney test and categorical variables were compared using the chi-square test or Fisher’s exact test as appropriate.

To adjust for potential baseline differences between the treatment and control groups, a propensity score analysis was performed to predict the conditional probability for an individual patient to receive a prompt introduction of normocaloric enteral nutrition [12]. The covariates included in the propensity score model were as follows: poor nutritional status before admission, temporarily closed loops, ischemic bowel disease, occurrence of refeeding hypophosphatemia and SOFA score at day 2. Inverse probability of treatment weighting (IPTW) was used for estimating the average treatment effect on time-to-event outcomes [12].

A multivariable logistic regression was then used to test the association between modalities of nutritional support (enteral nutrition vs. nil per os during the first week after OA/NPT, early vs. late enteral nutrition, normal vs. low caloric/protein intake) and the main study outcome, adjusting for covariates selected on an *a priori* basis: SAPS 2 at ICU admission, indication for OA/NPT (prophylactic OA vs. complicated surgery), maximal SOFA score and cumulated fluid balance at day 7. The covariates included in the model were known to be clinically relevant and particular attention was paid to interactions and multicollinearity [13].

A sample size of 150 patients was needed to limit potential sparse-data bias, assuming a 30% rate of poor clinical outcome with at least 5 dichotomous covariates incorporated in the model (including the exposure of interest) [14].

A p-value <0.05 was considered statistically significant. Statistical analyses were performed using XLSTAT 2015 for Windows (Addinsoft Paris, France) and Stata software (StataCorp, College Station, US).

## Results

### Characteristics of the Population

During the study period, 192 patients were treated by OA/NPT but 19 had an ICU length of stay <7 days after surgery. Moreover, two patients had incomplete nutrition-related data. Finally, 171 patients were included in the present study. Overall, poor clinical outcome was reported in 85 patients [delayed abdominal closure, N = 57 (33%), anastomotic leak, N = 30 (18%), intra-abdominal abscess, N = 20 (12%), abdominal dehiscence, N = 5 (3%)]. The main characteristics of the population are reported Table 2.

**TABLE 2:** Main characteristics of the population.

	Overall population N = 171 (100)	Good clinical outcome N = 86 (50)	Poor clinical outcome N = 85 (50)	*p*
Demographic and anthropometric data ‐ Age (years) ‐ Male sex ‐ Body mass index (kg/m^2^) ‐ Adjusted body weight (kg) Medical history ‐ SAPS 2 at ICU admission ‐ Charlson comorbidity index ‐ Poor nutritional status before admission	63 [50–70] 139 (81) 27 [24–31] 77 [66–84] 47 [36–64] 4 [1–5] 67 (39)	65 [51–73] 71 (83) 26 [24–30] 76 [64–81] 47 [35–63] 4 [1–5] 31 (36)	62 [48–67] 68 (80) 28 [24–31] 77 [67–85] 48 [37–66] 4 [1–6] 36 (42)	*0.043* *0.668* *0.312* *0.303* *0.601* *0.991* *0.398*
Type of initial surgery before OA/NPT ‐ Emergency surgery ‐ Traumatic surgery Time and indication for OA/NPT ‐ Prophylactic OA ‐ OA for secondary abdominal complication (*) o *Secondary peritonitis* o *Ischemic bowel disease* o *Abdominal Compartment Syndrome* o *Intra-abdominal bleeding* Number of surgical revisions before fascial closure Total duration of OA/NPT before fascial closure	117 (68) 48 (28) 115 (67) 33 (19) 22 (13) 21 (12) 18 (11) 3 [2–4] 6 [3–10]	63 (73) 29 (34) 65 (76) 10 (12) 8 (9) 9 (10) 8 (9) 2 [1–3] 4 [2–5]	54 (64) 19 (22) 50 (59) 23 (27) 14 (16) 12 (24) 10 (12) 4 [3–5] 10 [6–13]	*0.171* *0.098* *0.020* *0.011* *0.162* *0.467* *0.600* *<0.0001* *<0.0001*
Nutrition-related conditions over the first week after OA/NPT ‐ Use of enteral nutrition o Early enteral nutrition o Late enteral nutrition ‐ Use of parenteral nutrition ‐ Early enteral nutrition ‐ Use of parenteral omega-3 FA containing lipid emulsion ‐ Temporary contraindication of early enteral nutrition ‐ Occurrence of refeeding hypophosphatemia ‐ Mean caloric intake (kcal/kg/day) o *Underfeeding* o *Overfeeding* ‐ Mean protein intake (g/kg/day) o *Low protein diet* o *High protein diet* ‐ Mean glucose intake (g/kg/day) ‐ Mean lipid intake (g/kg/day)	94 (55) 35 (20) 59 (35) 165 (96) 134 (78) 44 (26) 107 (63) 39 (23) 19 [15–24] *46 (27)* *46 (27)* 0.9 [0.7–1.1] *19 (11)* *30 (18)* 2.1 [1.7–2.6] 0.7 [0.6–0.9]	56 (65) 22 (26) 34 (40) 81 (94) 66 (77) 20 (23) 53 (62) 26 (30) 19 [15–24] *26 (30)* *23 (27)* 0.9 [0.7–1.1] 8 (9) 15 (17) 2.1 [1.6–2.6] 0.7 [0.5–0.9]	38 (45) 13 (15) 25 (29) 84 (99) 68 (80) 24 (28) 54 (64) 13 (15) 19 [15–24] *20 (24)* *23 (27)* 0.9 [0.7–1.1] 11 (13) 15 (18) 2.1 [1.7–2.6] 0.7 [0.6–0.9]	*0.007* *0.096* *0.164* *0.099* *0.605* *0.456* *0.797* *0.020* *0.749* *0.323* *0.963* *0.994* *0.449* *0.972* *0.932* *0.439*
Other surgical and medical conditions over the first week after OA/NPT ‐ Cumulated fluid balance (L) ‐ Maximal SOFA score (without neurologic component) ‐ Use of CRRT ‐ Early use of mesh-fascial traction ‐ Early use of epidural analgesia	4 [0–7] 10 [8–12] 59 (35) 89 (52) 48 (28)	3 [0–7] 9 [8–11] 23 (27) 30 (35) 25 (29)	4 [0–8] 10 [7–12] 36 (42) 59 (69) 23 (27)	*0.307* *0.471* *0.032* *<0.0001* *0.770*
Other study outcomes ‐ Secondary VAP after OA/NPT ‐ In-hospital mortality ‐ Time before transit recovery ‐ Duration under mechanical ventilation ‐ ICU length of stay ‐ Hospital length of stay	46 (27) 31 (18) 7 [4–10] 9 [5–17] 20 [14–32] 34 [22–61]	26 (30) 8 (9) 7 [4–9] 8 [5–14] 19 [12–26] 32 [20–58]	20 (24) 23 (27) 7 [4–10] 12 [6–18] 22 [16–38] 38 [23–62]	*0.323* *0.003* *0.412* *0.009* *0.002* *0.066*

Results expressed as median [interquartile 25–75] or number (percentage).

### Association Between Nutrition-Related Conditions and Clinical Outcome

**FIGURE 1:**
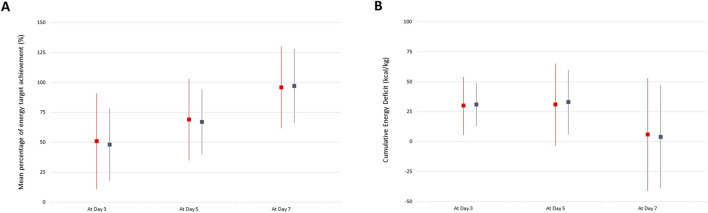
Mean percentage of energy target achievement **(A)** and Cumulative Energy Deficit **(B)** over time between patients with good (blue lines) vs. poor (red lines) clinical outcome.

In the non-adjusted population, the rate of poor clinical outcome was significantly lower in patients who received enteral nutrition versus those who remained nil per os (40% vs. 61%, *p = 0.007*). However, the rate of poor clinical outcome did not differ between patients who received early vs. late enteral nutrition (15% vs. 29%, *p = 0.165*). Moreover, the rate of delayed abdominal closure and/or intra-abdominal complication did not differ according to the mean percentage of energy target achievement and cumulative energy deficit over the first week after OA/NPT (Figure 1). Finally, there was no statistical difference between the mean protein, glucose and lipid intakes between patients with good vs. poor clinical outcome (Table 2).

**FIGURE 2:**
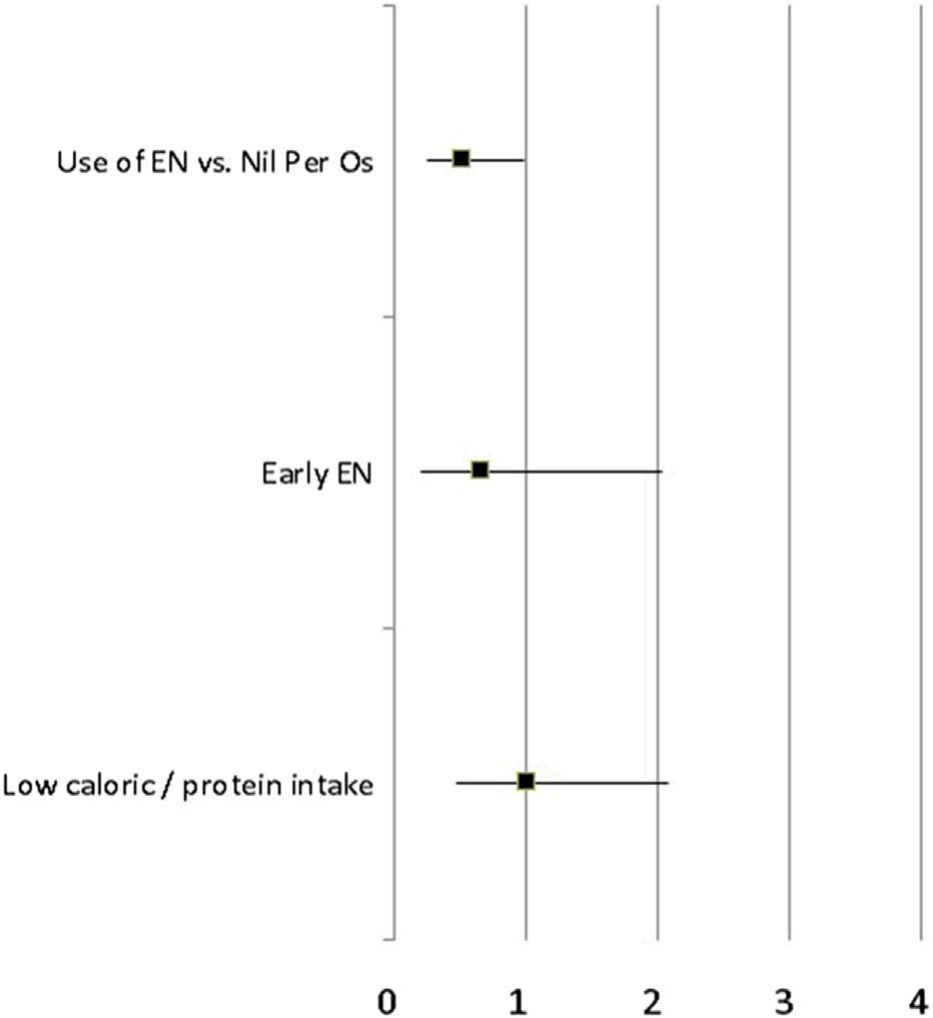
Covariate-adjusted OR of poor clinical outcome (delayed abdominal closure, secondary anastomotic leak, intra-abdominal abscess and fascial dehiscence) according to different modalities of nutritional support (use of EN vs. Nil per OS, early vs. late enteral nutrition, normal vs. low caloric/protein intake) in the propensity-matched population of critically ill patients with OA/NPT.

In the propensity-matched population, the covariate-adjusted OR of poor clinical outcome in patients who received enteral nutrition was 0.49 [95%CI: 0.25–0.98]. There was no other statistical association between modalities of nutritional support (early vs. late enteral or parenteral nutrition, normal vs. low caloric/protein intake) and the main study outcome (Figure 2). In the multivariable regression model, the prophylactic OA/NPT was the only covariate statistically associated with good clinical outcome [adjusted OR = 0.33 (95%CI: 0.15–0.72)].

The use of EN was statistically associated with a lower rate of secondary intra-abdominal complications [adjusted OR = 0.43 (95%CI: 0.21–0.87)] whereas there was no statistical difference regarding the rate of delayed abdominal closure [adjusted OR = 0.61 (95%CI: 0.31–1.20)]. When considering the time before abdominal closure as a continuous variable, the use of EN within the first week after OA/NPT was associated with a 7% (95%CI: 1%–15%) increase in the 28 days OA/NPT free days. There was no other statistical association between modalities of nutritional support and the number of days under OA/NPT before definitive fascial closure.

## Discussion

This study is one of the largest cohorts of critically ill patients with OA/NPT investigating the effect of the nutritional support on clinical outcome (abdominal wall closure rate or fistula formation). Our results suggested that the use of EN during the acute phase (i.e., the first 7 days) following surgery was associated with a shorter duration under OA/NPT and a lower rate of secondary intra-abdominal complications. However, neither the time of introduction nor the amounts of caloric and protein intake were associated with clinical outcome.

In this concern, conflicting results are reported in the literature. On the one hand, Byrnes et al. suggested that early EN was associated with a longer time with OA/NPT (7.1 vs. 3.4 days), although this underpowered study was impaired by a strong selection and interpretation bias [8]. This result was not confirmed by Dissanaike et al. who reported no effect of immediate EN (within 36 h) on abdominal closure rate [6]. On the other hand, Collier et al. were the first to report earlier fascial closure rates with the initiation of EN before day 4 after injury [5]. However, this study was impaired by the presence of multiple confounding variables. Accordingly, the Western Trauma Association (WTA) also reported higher fascial closure rates (OR = 5.3; *p < 0.01*), decreased complication rates (OR = 0.46; *p = 0.02*), and decreased mortality (OR = 0.30; *p = 0.01*) in patients without intestinal injury who received EN before the closure of the abdomen [7]. Different outcomes were reported in the sub-group analysis of patients with bowel injuries, suggesting the presence of intestinal injury and the ICU-related conditions should be taken into account before starting nutritional support. In this context, our results also suggested an independent association between the etiology of OA and clinical outcome.

Questions such as time for introduction (early vs. late EN) and amount of enteral nutrition (goal tube feeds vs. trophic feeds) remained to be investigated among patients with OA/NPT. In this regard, our results found no statistical association between caloric and protein intake and clinical outcome. It is thus unlikely that the benefit of enteral feeding derives from a better macronutriment intake. Our results are thus in accordance with former studies suggesting similar outcomes between permissive underfeeding to those with standard enteral feeding [15–18]. The association with clinical outcome may be explained by the other advantages of EN, such as improving vascular flow, reducing bowel edema, maintaining bowel mucosal integrity and lessening septic complications [19–21]. Accordingly, our study may suggest that i) a prompt introduction of EN at trophic levels after OA/NPT appears to be beneficial in the absence of contraindication and ii) the caloric/protein intake should be progressively increased up given the risk of refeeding syndrome in the early phase of acute illness.

However, our study was underpowered for an adequate assessment of outcome according to the protein delivery, with very few patients with low (i.e. < 0.5 g/kg/day) protein intake. Recent studies suggested a strong association between negative nitrogen balance, muscle depth loss and clinical outcome of critically ill patients [22]. Preserving a positive nitrogen balance may be of paramount importance amongst patients with OA/NPT, where a negative nitrogen balance is a common occurrence [6]. Of note, only 18% of patients reached the adequate protein requirement, which should be in the range of 1.3–2.5 g/kg/day in this specific population. Whether the use of specific customized high-protein concentration mixtures may improve clinical outcome in this specific population deserves further studies [23].

Several limitations should be reported. The main limitation relied on the retrospective design that could lead to selection and interpretation bias. Although the multivariable regression in an IPTW propensity-matched population allowed reducing the effects of confounding covariates between treatment groups, there is still a possibility that the observed association is due to unrecorded or unobserved confounders (specifically contra-indication for oral feeding). However, there was no statistical association between contra-indication of oral feeding and clinical outcome. Moreover, there is no consensus statement on the definition of delayed abdominal closure, arbitrarily fixed at 7 days in accordance with previous studies [24]. Finally, the large majority of patients had adequate caloric and protein intakes, limiting the sample size for subgroup analysis (underfeeding regimen vs. overfeeding and low vs. high protein intake).

## Conclusion

In critically ill patients with OA/NPT, the use of enteral feeding within 7 days after surgery was associated with better clinical outcome. The time of introduction of EN and the caloric/protein intakes were not associated with abdominal wall closure rates or fistula formation, although our study was underpowered to assess the effect of high protein intake. Further studies are mandatory to better define the adequate energy and protein intake during the acute phase after OA/NPT.

## Data Availability

The raw data supporting the conclusions of this article will be made available by the authors, without undue reservation.

## Abbreviations

OA/NPT, open abdomen with negative pressure therapy; IPTW, Inverse probability of treatment weight; OR, Odds ratio; ICU, Intensive Care Unit; EN, Enteral nutrition; PN, Parenteral nutrition; RFS, Refeeding syndrome; BMI, Body mass index; ABW, Adjusted body weight; SAPS, Simplified Acute Physiology Score; SOFA, Sequential Organ Failure Assessment; CRRT, Continuous renal-replacement therapy; VAP, Ventilator-acquired pulmonary infections.
